# Hydrogenase Mediated Biosynthesis of Catalytically Active Cu Nanoparticles

**DOI:** 10.1002/smll.202500210

**Published:** 2025-07-14

**Authors:** Natalie Byrd, Christopher Egan Morriss, Joseph Parker, Rongsheng Cai, Elliott J. Nunn, Jessica H. van Wonderen, Jennifer S. Cavet, Fabio Parmeggiani, Richard L. Kimber, Jeffrey A. Gralnick, Thomas A. Clarke, Sarah J. Haigh, Jonathan R. Lloyd

**Affiliations:** ^1^ Department of Earth and Environmental Sciences The University of Manchester Manchester M13 9PL UK; ^2^ Department of Materials University of Manchester Oxford Road Manchester M13 9PL UK; ^3^ Lanzhou Institute of Chemical Physics Chinese Academy of Sciences Lanzhou 730000 China; ^4^ Department of Chemistry The University of Manchester Manchester M13 9QQ UK; ^5^ School of Biological Sciences University of East Anglia Norwich Research Park Norwich NR4 7TJ UK; ^6^ School of Biological Sciences Faculty of Biology Medicine and Health University of Manchester Manchester M13 9QQ UK; ^7^ Department of Chemistry Materials and Chemical Engineering “G. Natta” Politecnico di Milano Milan 20131 Italy; ^8^ Department of Plant and Microbial Biology University of Minnesota‐Twin Cities St. Paul MN 55108 USA

**Keywords:** bioreduction, biosynthesis, click chemistry, Cu nanoparticle catalyst, hydrogenase, nanobiotechnology

## Abstract

*Shewanella oneidensis* MR‐1 can biosynthesize cell‐supported Cu‐nanoparticles (CuNPs), via the bioreduction of Cu(II)_(aq)_, with excellent catalytic activity for click chemistry reactions. However, enzymatic mechanisms underpinning Cu(II) bioreduction were unclear. Here, the oxidation of hydrogen as electron donor was essential for Cu(II) bioreduction by *S. oneidensis* and hydrogenase deletion mutants were used to demonstrate the critical role of the periplasmic [NiFe] hydrogenase, HyaB. Wild type (WT) cultured cells coupled hydrogen oxidation to biosynthesis of Cu(0)/Cu(I)‐NPs within the periplasm (identified using XRD and TEM with SAED, EDS, EELS); *ΔhyaB* mutants did not produce CuNPs. Biosynthesized CuNPs were catalytically active for the cycloaddition of methyl azidoacetate and 1‐hexyne, confirming the potential for microbial revalorization of Cu(II)‐containing wastewaters, by forming catalytically active nanomaterials. Identifying HyaB, as a key mediator for Cu(II) reduction in *S. oneidensis* is an important first step towards developing industrial bioprocesses for Cu(II) recovery and CuNP synthesis, offering a template for improvements using engineering biology. Interestingly, *c*‐type cytochromes, critical for reduction of other metals, were unable to fully reduce Cu(II)_(aq)_ in vivo despite being capable of Cu(II) reduction under in vitro conditions. In fact, Cu inhibited outer membrane cytochrome mediated reduction of Pd(II), and this may impact bioreduction of mixed metal solutions/effluents.

## Graphical abstract

*Shewanella oneidensis* MR‐1 biosynthesized cell‐supported, catalytically active Cu‐nanoparticles (CuNPs). Hydrogen is the only electron donor that supports Cu(II) bioreduction to CuNPs, with hydrogenase deletion mutants indicating involvement of the periplasmic [NiFe] hydrogenase, HyaB. Bio‐CuNPs comprised Cu(0)/Cu(I) and catalyzed the cycloaddition of methyl azidoacetate and 1‐hexyne. This is an important first step toward sustainable industrial bioprocesses for Cu(II) recovery and CuNP biosynthesis.

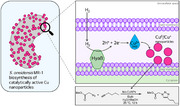

## 1 Introduction

*Shewanella oneidensis* MR‐1 is a widely studied model metal‐reducing bacterium, able to reduce a range of metals, including Cr(VI), Tc(VII), Pd(II), U(VI), Pu(VI/V) under anaerobic conditions.^[^ ^1^, ^2^, ^3^, ^4^, ^5^ ^]^ *Shewanella* species are prominent in (sub)terrestrial and aquatic ecosystems, and their role in global metal biogeochemical cycling is well documented.^[^ ^6^, ^7^ ^]^ *S. oneidensis* MR‐1 was recently shown to bioreduce Cu(II), an essential element for life, but also a toxic contaminant when present in excess.^[^ ^8^, ^9^ ^]^ Cu is also central to the green energy transition; required for decarbonization, renewable power generation (e.g., in wiring for electrical transmission), and as a catalyst for production of valuable organic compounds (e.g., pharmaceuticals).^[^ ^10^, ^11^, ^12^ ^]^ Future Cu demand is expected to surge, challenging Cu supply, and accordingly methods for green biorecovery of Cu from unconventional resources will become essential to ensure resource efficiency.^[^ ^13^, ^14^ ^]^ The metal‐reducing ability of subsurface bacteria, like *S. oneidensis* MR‐1, could be harnessed for cost effective and environmentally benign solutions for Cu/metal biorecovery from unconventional resources, key examples of which include mining and landfill effluents, industry waste waters, and e‐waste reprocessing effluents.^[^ ^15^, ^16^ ^]^ Utilizing metal‐reducing bacteria to bioreduce soluble, high‐valence metals directly results in the precipitation of valuable, cell‐supported metal nanoparticles (NPs). Not only is this useful for biorecovery, but bio‐NPs have many applications, including as nano‐sized catalysts, e.g., CuNP catalysts are used in click chemistry reactions.^[^ ^17^, ^18^ ^]^ Additionally, bacterial NP synthesis offers a scalable biotechnology that can be fine‐tuned, e.g., to enhance recovery using synthetic biology.^[^ ^17^, ^18^, ^19^, ^20^ ^]^ This presents opportunities for development of microbial metal‐bioreduction technologies that can simultaneously de‐contaminate and valorize effluents, promoting the circular economy.

*S. oneidensis* can produce catalytically active CuNPs via bioreduction, in addition to other valuable NPs including Se, Au, Ag, and Pd.^[^ ^9^, ^21^ ^]^ However, Cu(II) bioreduction mechanisms in this model organism are poorly understood which, combined with Cu toxicity, presents challenges to the optimization and industrial scale‐up of Cu biorecovery and CuNP synthesis.^[^ ^9^, ^22^, ^23^ ^]^ Understanding the underpinning mechanisms in *S. oneidensis* MR‐1 is essential to optimize biorecovery/bioreduction, and in turn fine‐tuning the properties of nanoparticle products (e.g., size, shape, cellular location), through engineering biology approaches.^[^ ^18^, ^19^, ^24^, ^25^, ^26^ ^]^ Previous work highlighted that the *c‐*type cytochrome based Mtr pathway commonly implicated in reduction of other high valence metals (e.g., Cr(VI), and U(VI)^[^ ^1^, ^2^, ^27^ ^]^), did not mediate full Cu(II) bioreduction and CuNP synthesis in *S. oneidensis* MR‐1.^[^ ^9^ ^]^ Besides *c*‐type cytochromes, some metal bioreduction pathways are also known to be facilitated by hydrogenases including Pd(II), Au(III) and Tc(VII) bioreduction by *S. oneidensis* MR‐1, and other organisms,^[^ ^28^, ^29^, ^30^, ^31^, ^32^, ^33^, ^34^ ^]^ but these have not been studied in the context of Cu(II) bioreduction. The genome of *S. oneidensis* encodes two periplasmic hydrogenases, which are expressed under anaerobic conditions.^[^ ^35^ ^]^ Our focus was to investigate the potential role of hydrogenases in Cu(II) reduction by *S. oneidensis* MR‐1, and the interplay between Cu(II) and *c*‐type cytochromes. The use of different electron donors (H_2_ and lactate, or pyruvate fermentation) was explored in Cu(II) bioreduction experiments, supported by in vivo and in vitro experiments using hydrogenase deletion mutants and purified *c*‐type cytochrome (MtrC), respectively. CuNPs synthesized via a hydrogenase mediated pathway in whole cells were characterized and tested for their catalytic activity (in a model click chemistry reaction), confirming a novel, green hydrogen‐mediated biosynthesis route for the conversion of soluble Cu(II) to useful nanocatalysts.

## 2 Results and Discussion

### 2.1 CuNP Synthesis with H_2_ as Electron Donor

**Figure 1:**
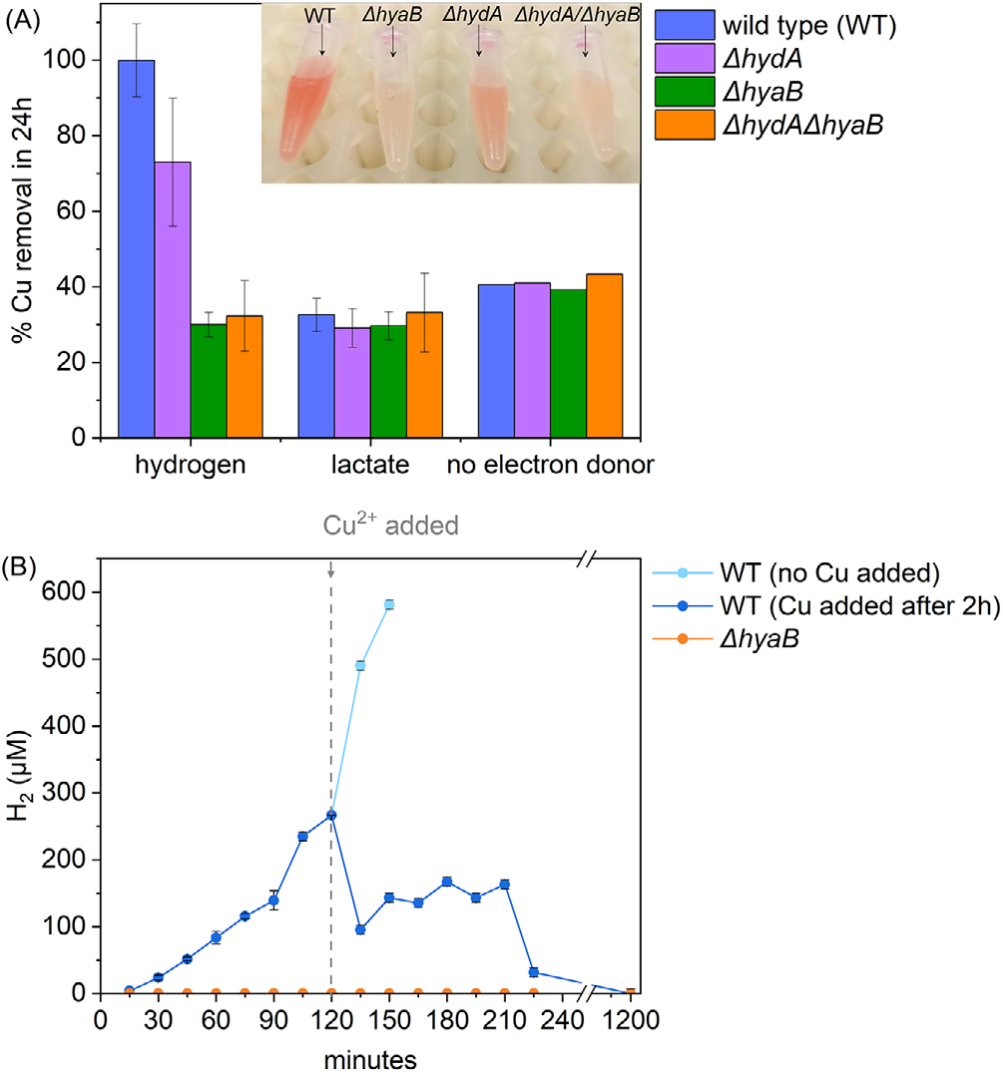
Cu removal data showing H_2_ is utilized to support Cu(II) bioreduction in S. oneidensis. A) Inductively coupled plasma mass spectrometry (ICP‐MS) data for % Cu(II) removal at 24 h by wild type S. oneidensis and various hydrogenase deletion mutant strains and electron donors. Inset: picture showing colour of reaction of cell cultures after 24 h incubation with H_2_ as electron donor B) Gas chromatography (GC) data for headspace monitoring experiments incubated with 10 mm pyruvate showing H_2_ generation (no Cu; 0–120 min) and consumption after 100 µm Cu(II) was added (120–1200 min) for wild type and ΔhyaB cells. Error bars indicate 1σ on triplicate experiments, except no electron donor controls in (A) which were not replicated.

Washed cells of *S. oneidensis* MR‐1 were incubated at pH 7 in bicarbonate buffer, with 100 µm Cu(II) and either lactate (10 mm) or under a 100% H_2_‐headspace as the sole electron donor. After 24 h, bottles supplemented with H_2_ changed from colourless to pink, consistent with Cu(II) bioreduction,^[^ ^9^ ^]^ accompanied by 98% removal of aqueous Cu(II) (**Figure** **1A**; Figure S1A, Supporting Information). In contrast, no Cu(II) removal, solid precipitation or color change was observed in no cell controls, indicating H_2_ did not abiotically reduce Cu(II). Cells supplemented with lactate as electron donor did not turn pink and only 26.9% Cu(II) removal was measured (Figure 1A). Lactate was not metabolized (Figure S1, Supporting Information) and therefore was not used as an electron donor to support Cu(II) bioreduction. Data from the no electron donor control showed 48.9% Cu(II) removal, likely by biosorption, and it is possible that in the lactate‐supplemented experiments (displaying lower levels of biosorption) some Cu‐lactate complexes formed, modestly inhibiting Cu(II) removal from solution (Figure 1A; Figure S1A, Supporting Information).^[^ ^36^ ^]^

### 2.2 Cu(II) Bioreduction Mechanisms in *S. oneidensis* MR‐1

The clear indication that H_2_ was required for *S. oneidensis* to bioreduce Cu(II) and form CuNPs suggested involvement of hydrogenase enzymes. Accordingly Cu(II) bioreduction experiments were performed using hydrogenase deletion mutants; *ΔhydA* (lacks [FeFe] hydrogenase, HydA)*, ΔhyaB* (lacks [NiFe] hydrogenase, HyaB)*, ΔhydA/ΔhyaB* (lacks both HydA and HyaB).^[^ ^35^ ^]^ Washed cells of *S. oneidensis* (wild type, or hydrogenase deletion mutants) were incubated at pH 7 in bicarbonate buffer with 100 µM Cu(II) with H_2_ in the headspace as the sole electron donor. Bottles containing either wild type (WT) or *ΔhydA* cells turned pink within 24 h, and Cu(II) removal was 100% and 73%, respectively (Figure 1A). Both systems displayed enhanced Cu(II) removal compared to no‐electron donor controls (40.6% and 41%, respectively), suggesting bioreduction of soluble Cu(II) to insoluble Cu(0) or Cu(I) had occurred. In contrast, bottles containing [NiFe] hydrogenase deletion mutants, *ΔhyaB* and *ΔhydA/ΔhyaB*, showed no color change (after 72 h incubation), with Cu(II) removal at 30% and 32.3%, respectively by 24 h; within error of corresponding no donor controls (39.2% and 43.4%). Incomplete Cu(II) removal (73.0 ± 16%) was noted in the *ΔhydA* system, and could indicate that HydA (H_2_ forming, [FeFe] hydrogenase) has a minor role in supporting CuNP biosynthesis. These results show that the [NiFe] hydrogenase, HyaB, is essential for Cu(II) bioreduction by *S. oneidensis*, similarly to other metals, e.g., Tc(VII) and Pd(II),^[^ ^4^, ^5^, ^34^ ^]^ with purified HyaB shown to directly reduce Tc(VII) to Tc(IV).^[^ ^34^ ^]^ Surprisingly, previous suggestions that Cu(II) is a selective inhibitor of hydrogenase enzymes, based on [NiFe] hydrogenases from other organisms,^[^ ^37^ ^]^ do not appear to be true for *S. oneidensis*.

### 2.3 Hydrogen Metabolism and Cu(II) Bioreduction

To confirm that H_2_ metabolism supported Cu(II) bioreduction and explore the involvement of HyaB, hydrogen headspace monitoring was performed during experiments under pyruvate‐fermenting (and then Cu(II)‐reducing) conditions. Knowing that in the absence of an electron acceptor *S. oneidensis* MR‐1 ferments pyruvate to H_2_ via HyaB,^[^ ^35^ ^]^ we incubated wild type or *ΔhyaB* cells with 10 mm pyruvate only and monitored H_2_ levels in the headspace over 2 h. In the wild type (WT) experiment, we observed continuous hydrogen production reaching 226.8 ± 0.4 µm H_2_ in 2 h. No H_2_ was detected in any samples from the *ΔhyaB* experiment (Figure 1B), confirming that HyaB (bidirectional H_2_ forming and oxidizing [Ni–Fe] hydrogenase) was expressed and active in WT cells, but not in the *ΔhyaB* cells. After 2 h of preincubation with pyruvate and H_2_ generation, 100 µm Cu(II) was spiked into the bottles and the headspace was monitored for a further 2 h, and then again at 20 h. After 20 h, in the WT system all H_2_ had been consumed and vials had turned pink, indicating H_2_‐driven Cu(II) bioreduction had occurred via HyaB. When no Cu(II) was added to the WT system, H_2_ continued to accumulate continuously beyond 2 h (Figure 1B), confirming that the Cu(II) bioreduction was consuming the H_2_ that had been generated. By 20 h, after Cu(II) addition, in WT systems no H_2_ was detected suggesting pyruvate fermentation had stopped. Potential explanations include pyruvate depletion, or, that Cu may be slowly deactivating HyaB. Based on the current work, HyaB seems to tolerate Cu exposure in resting cell experiments with H_2_ as electron donor. Future investigations to understand the sensitivity of HyaB to Cu would be useful.

These data suggest that Cu(II)‐reducing activities may be optimized by including a pyruvate‐fermenting pre‐incubation step. For example, it was recently reported that pre‐incubating resting cells of *S. oneidensis* with pyruvate for 2 h resulted in H_2_ generation, increased Pd(II) reduction rates and upregulation of transcriptional levels of both HyaB (*hyaAB*) and the Mtr pathway (*cymA, omcA*, and *mtrCAB*). This effect was not observed during similar “pre‐incubations” with lactate or formate suggesting a key role for pyruvate in signaling and transcriptional regulation of metal‐reducing enzymes.^[^ ^38^ ^]^ Potentially, systems could be designed to “self‐generate” H_2_ from pyruvate, with H_2_ supply localized to cells for more controlled bio‐NP synthesis, upregulation of metal‐reducing systems, enhanced bioreduction rates, and improved cell viability.^[^ ^38^ ^]^ Although we did not explore H_2_ generation via the HydA (unidirectional [Fe–Fe] hydrogenase, hydrogen forming), it is not relevant as a direct metal reductase and previous work suggests it is much less active than HyaB under our experimental conditions.^[^ ^34^, ^35^, ^39^ ^]^ However, in an environmental context, under different conditions, HydA may be important for supporting metal bioreduction by local pH buffering (via H^+^ consumption) and/or by regenerating H_2_ to feedback into HyaB.^[^ ^35^, ^39^ ^]^

### 2.4 Cu(II) Interactions with Outer Membrane Cytochromes

Additional experiments were conducted to explore why CuNP synthesis does not proceed via lactate‐driven outer membrane cytochromes. In all systems, rapid removal of Cu(II) (20–30%) was observed in the first 15 min, presumed to be via biosorption. Sustained/enhanced Cu(II) removal was only observed in the H_2_ supplemented experiments, with Cu removal plateauing after 2 h in the non‐metabolically active systems (lactate, no donor) (Figure 1A; Figure S1, Supporting Information). To determine whether non‐metabolic Cu(II) reduction had occurred, or if removal was solely due to Cu(II) biosorption, we reacted aliquots of cell cultures challenged with Cu(II) (under N_2_ atmosphere) for 24 h with bathocuproine ligand solution. Bathocuproine has high specificity for Cu(I) and forms a characteristic orange complex.^[^ ^40^ ^]^ After one minute, both aliquots were visibly orange (Figure S2, Supporting Information) implying cells reduced a portion of Cu(II) to Cu(I) without the requirement for an added electron donor (Figure S1B, Supporting Information), i.e., non‐metabolically, by using residual reducing potential from biomass production. Our findings are in agreement with previous work showing that under anaerobic conditions (in the absence of H_2_) *S. oneidensis* performed rapid partial reduction of Cu(II)‐EDTA to Cu(I) (32.5% Cu removal in ≤10 min), which was linked to outer membrane cytochromes (OMCs).^[^ ^41^ ^]^

**Figure 2:**
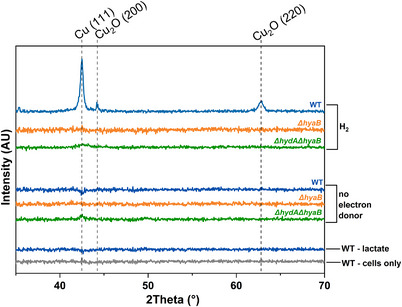
X‐ray powder diffraction data collected from washed cell pellets from bioreduction experiments. Annotations in black writing (right hand side) describe the electron donor supplied and coloured annotations (inset) correspond to lines of the same colour and describe the strain used to generate the sample.

Here, we postulate that partial Cu(II) reduction in the non‐metabolically active systems was driven “passively” by contact between Cu(II) and reduced OMCs.^[^ ^1^, ^5^, ^41^ ^]^ We tested this using MtrC as a model OMC by reacting 100 µm Cu(II) with 0.9 µm of purified MtrC (reduced with sodium dithionite under anaerobic conditions), in vitro. MtrC was re‐oxidized rapidly (in less than 3 min; Figure S3, Supporting Information), demonstrating that contact with reduced OMCs can facilitate rapid Cu(II) reduction. It has been established that whole cells with surface exposed MtrC (i.e., anaerobic resting cells) exchange electrons similarly to the purified MtrC protein.^[^ ^42^ ^]^ Our data showing the presence of Cu(I) and rapid Cu(II) induced re‐oxidation of purified, ferrous MtrC (Figure 1; Figures S3 and S1, Supporting Information) support the suggestion that reduced OMCs mediated a degree of Cu(II) reduction in our experiments. However, it remains perplexing that while OMC‐mediated Cu(II) bioreduction is clearly feasible, OMCs expressed in vivo do not readily facilitate complete Cu(II) bioreduction to Cu(0) for CuNP synthesis (Figure S1, Supporting Information; **Figure** **2**; Figure S3, Supporting Information). Currently, reasons for this are unclear and warrant further investigation; potential explanations include: a) the Cu‐MtrC interaction, e.g. binding^[^ ^43^ ^]^ of Cu(II) or Cu(I), disrupts the electron transfer chain, potentially altering the OMC structure in vivo b) reduction to Cu(I) impacts cellular function, e.g., via toxicity^[^ ^44^ ^]^ and/or c) Cu(I) is inaccessible for further reduction, e.g. due to chelation, preventing CuNP nucleation. We emphasize that OMCs of metal‐reducing bacteria can be critical for producing cell‐bound extracellular nanoparticles (e.g., for Pd and other metals) that attribute the highest catalytic activity.^[^ ^26^ ^]^

Understanding whether Cu disrupts/alters OMC activity is vital as this could impair extracellular bio‐NP synthesis from, e.g., mixed‐metal effluents containing Cu, like from waste electronic equipment reprocessing.^[^ ^45^, ^46^ ^]^ To probe whether Cu impairs extracellular electron transfer (EET) to other metals, cells were first incubated for 24 h with 100 µm Cu(II) and 10 mm lactate before being challenged with 180 µm Pd(II). After Pd(II) addition and further 72 h incubation, no evidence of *c*‐type cytochrome mediated Pd(II) reduction^[^ ^26^ ^]^ was observed (no color change or Pd(0)‐NPs detected by X‐ray diffraction (XRD); Figure S4, Supporting Information). This suggested that exposure of cells to Cu(II) impacted Pd(II) bioreduction by the OMCs, preventing PdNP synthesis. Clearly more in‐depth study is necessary to fully understand the Cu‐OMC interactions, however exposure to Cu does appear likely to impair the ability of *Shewanella* cells to perform EET by OMCs.

### 2.5 Characterization of Mixed Cu(I)/Cu(0) Biosynthesized Nanoparticles

XRD analysis was used to examine the crystalline structure of the CuNPs produced under different electron donor conditions. Crucially, only H_2_ amended WT samples possessed the peak at a 2*θ* value of 43.3° corresponding to the {111} planes of face‐centered cubic Cu(0), demonstrating complete Cu(II) bioreduction to Cu(0) (Figure 2, **Table** **1**). In addition, peaks at 2*θ* values of 42.8° and 61.4° corresponding to the {200} and {220} planes of Cu_2_O were also observed in these WT cells, indicating crystalline Cu(I) oxide had formed.^[^ ^47^, ^48^, ^49^ ^]^ Samples from H_2_‐supplemented, *ΔhyaB* and *ΔhydA/ΔhyaB* experiments ([NiFe] hydrogenase, HyaB, was removed), did not produce any XRD peaks, supporting our observation that HyaB is crucial for full Cu(II) bioreduction and CuNP synthesis in *S. oneidensis* (Figure 2). The no electron donor controls and lactate supplemented samples did not produce any XRD peaks, indicating no crystalline Cu species had formed due to insufficient Cu(II) bioreduction (Figures 2 and 1A). Scherrer analysis based on XRD peak measurements for the WT with H_2_ experiment (Figure 2, Table 1) determined that Cu‐containing crystallite sizes were nanoparticulate in the range 12–27 nm. Interestingly, these data indicated a bimodal particle size distribution indicating that smaller particles (<20 nm) were mainly Cu_2_O and larger particles (> 20 nm) were mainly Cu(0) (Figure 2, Table 1). Selected samples underwent further analysis by transmission electron microscopy (TEM).

**Table 1:** Values obtained from indexing XRD peaks and SAED patterns collected from solids in Cu(II) bioreduction experiments using H_2_ as an electron donor and S. oneidensis MR‐1 wild type cells (“WT”). Reference values from the International Centre for Diffraction Data (ICDD) are included for comparison. A dash indicates that no peak/diffraction pattern was obtained during analysis.

	d‐spacing [nm]							
	Cu(0)					Cu_2_O		
	{111}	{200}	{220}	{311}	{331}	{111}	{200}	{220}
ICDD value	0.209	0.179	0.128	0.109	0.083	0.247	0.214	0.151
WT, H_2_ (XRD; Figure 2)	0.205	–	–	–	–	–	0.214	0.154
WT, H_2_ (SAED; Figure 3)	0.209	0.181	0.128	0.109	0.082	0.247	–	–

**Figure 3:**
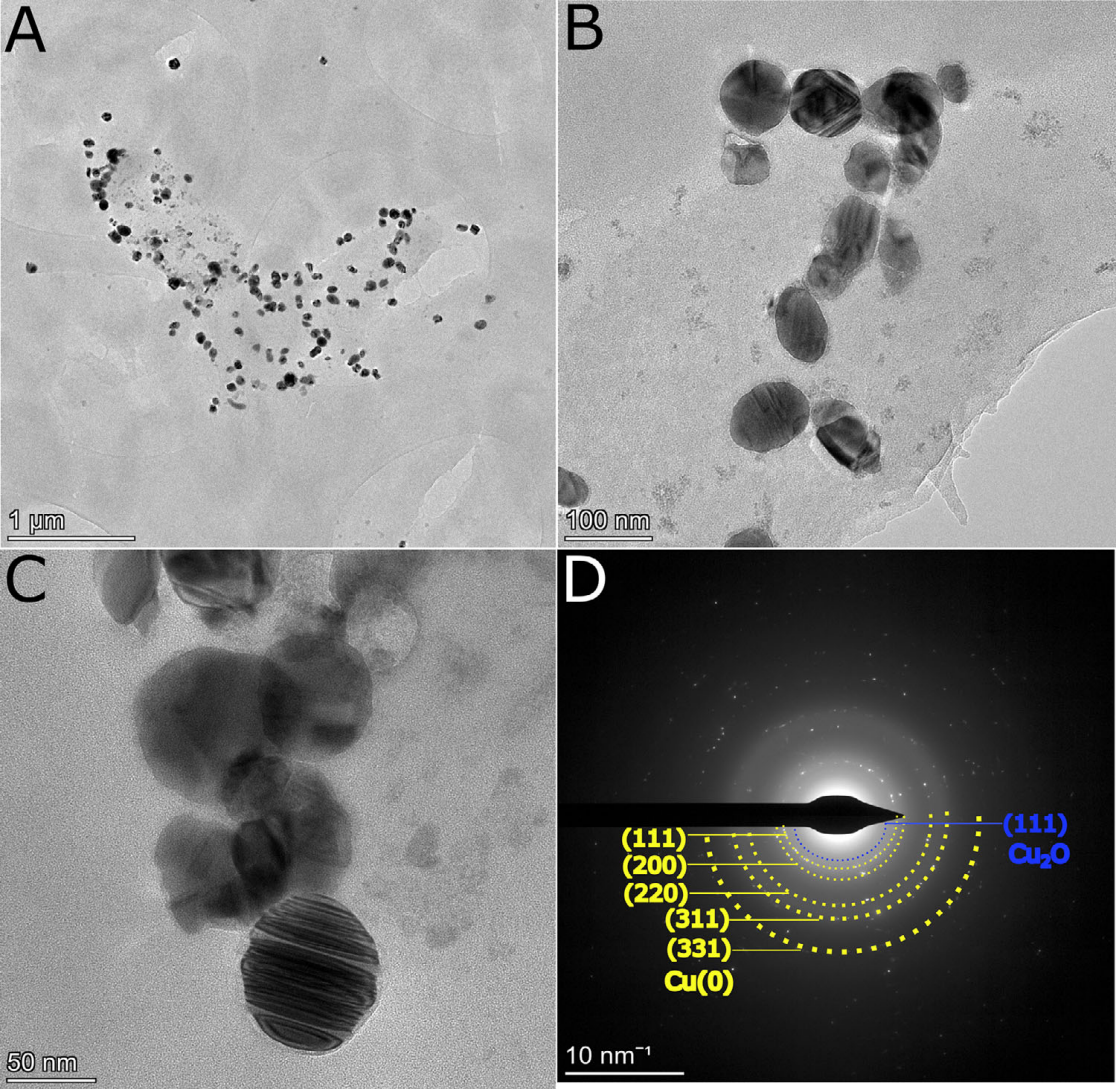
TEM images of whole mount samples of CuNPs synthesized by S. oneidensis MR‐1; A) shows whole cells populated with CuNPs, B,C) higher magnification images showing NP morphologies and crystal defects, and D) SAED pattern, with rings used for indexing labeled in yellow for Cu(O) and blue for Cu_2_O.

Whole mount TEM analysis of the WT experiments confirmed nanoparticles had formed (**Figure** **3**). Selected area electron diffraction (SAED) analysis (Figure 3D, Table 1) of the CuNPs from the WT experiment (with H_2_ as electron donor), was consistent with the XRD data (Figure 2) with d‐spacings that corresponded to both cubic Cu(0) and cubic Cu(I)_2_O (Table 1). Generally, CuNPs were well‐dispersed across the bacterial cell with a mean particle diameter of 29.7 nm (based on measurement of TEM images for 680 random nanoparticles from 3 replicate samples). However, two distinct populations were observed in the size distribution (Figure S5, Supporting Information); 62% of the particles had diameters ≤25 nm and a peak diameter of 10 nm, while the remaining fraction of particles were larger, in the range 25–100 nm. High magnification TEM images of the bio‐CuNPs reveal the larger nanoparticles are highly crystalline, albeit containing crystal defects such as stacking faults and nanotwinning (Figure 3B,C) features known to alter the physical, chemical and mechanical properties of nanocrystalline metals. In terms of biotechnological applications, defects change coordination environments and electronic configurations of the surface that may be beneficial, e.g., for controlling/enhancing catalytic activity.^[^ ^50^, ^51^, ^52^, ^53^ ^]^

**Figure 4:**
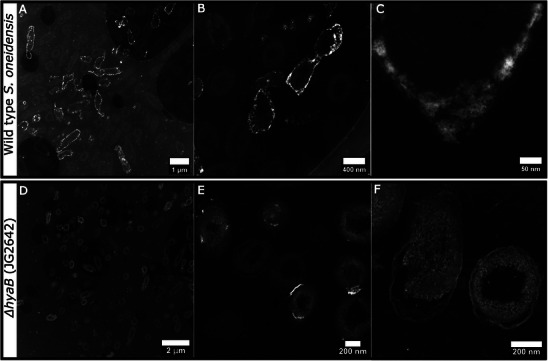
Thin section HAADF STEM images showing CuNPs present in the periplasm of wild type A–C) S. oneidensis MR‐1 cells, compared to ΔhyaB cells D–F), where no CuNPs can be seen (in these ΔhyaB samples electron dense areas are Pb, from post‐sectioning stain required to improve imaging contrast of light element cellular features). Samples were taken at the experimental end point (24 h).

To examine/compare nanoparticle formation and localization, samples from WT and *ΔhyaB* experiments were resin embedded and ultrathin sectioned. In WT samples, thin‐section high angle annular dark field (HAADF) scanning TEM (STEM) images revealed nanoparticles were abundant in the periplasm, visible as the bright (higher atomic number) areas tracing the outlines of cells (**Figure** **4A,C**) and were situated between distinguishable inner and outer membranes (Figure 4C). This aligns with the location of periplasmic hydrogenase enzymes, further confirming their involvement in Cu(II) bioreduction. Thin section TEM imaging also showed some CuNPs extended extracellularly (Figure 4A), likely due to continued bioreduction and CuNPs nucleating from the periplasm outward. In contrast, no CuNPs were observed with *ΔhyaB* cells in agreement with XRD, as HyaB was not present to facilitate Cu(II) bioreduction. Large electron dense precipitates were observed in *ΔhyaB* thin sections from the Pb‐citrate post‐resin stain, confirmed as Pb from EDS analysis (Figure 4E; Figure S7, Supporting Information). STEM EDS analysis also indicated non‐crystalline Cu present near the outer membrane, in agreement with our observations of Cu removal by cells via Cu(II) biosorption and non‐metabolic reduction to Cu(I) (Figure S7, Supporting Information).

**Figure 5:**
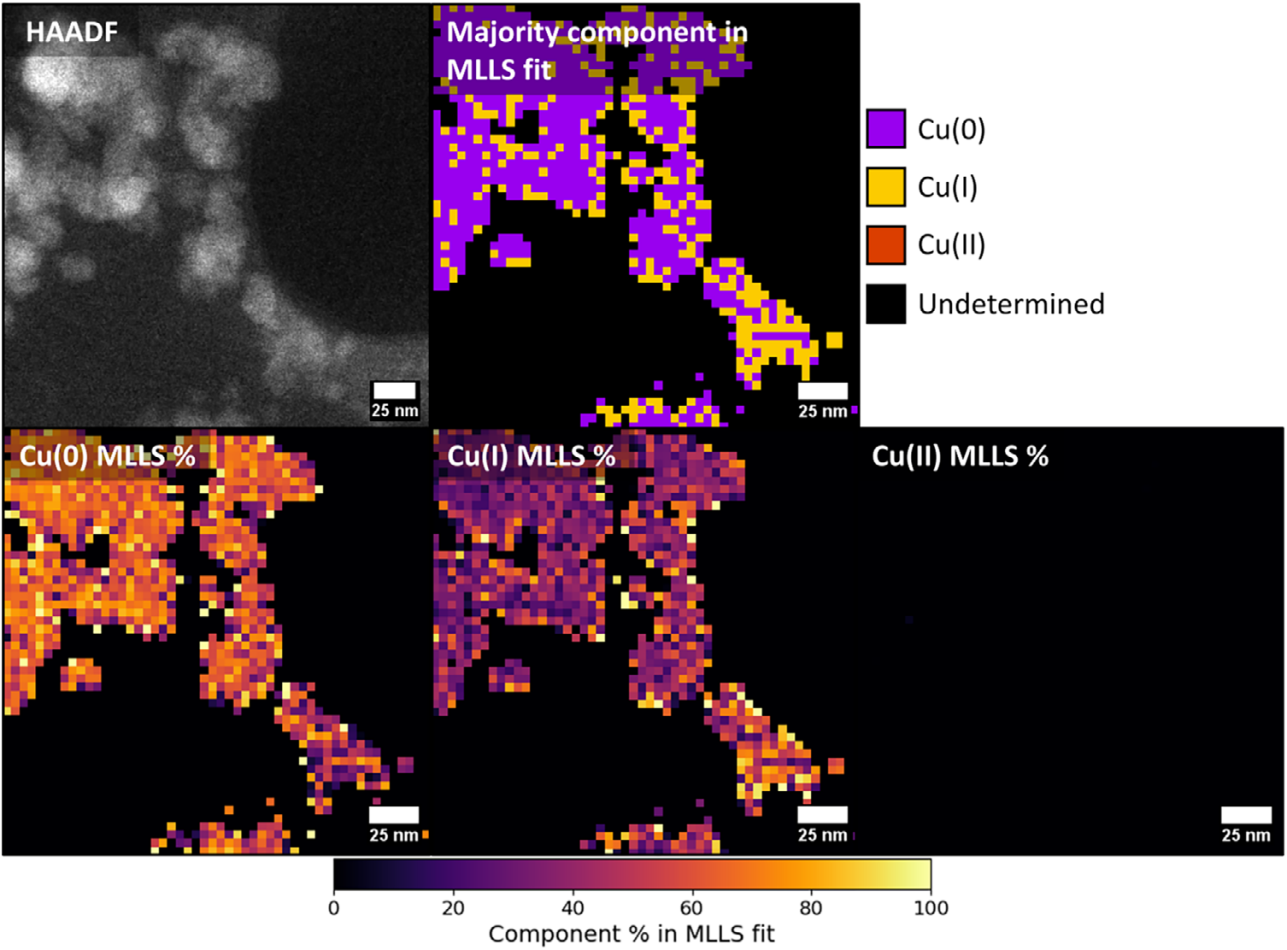
HAADF image of CuNPs and corresponding EELS maps showing species distributions calculated by MLLS fitting against reference spectra (shown in Figure S8, Supporting Information).

All diffraction data (TEM‐SAED, XRD) from WT generated CuNPs suggested nanoparticles comprised a mix of Cu(0) and Cu(I)_2_(O), therefore STEM electron energy loss spectroscopy (EELS) analysis was performed to further examine their composition. Spectra collected from nanoparticles at the Cu L_2,3_‐edge underwent multiple linear least squares (MLLS) fitting to reference spectra for Cu(0), Cu(I) or Cu(II) that indicated that samples did not contain any Cu(II) and comprised only reduced Cu species (**Figure** **5**; Figure S6, Supporting Information). The best fit for the CuNPs, informed by XRD and SAED, indicated that nanoparticles comprised mostly metallic Cu(0) with some Cu(I) suggested to be present on the exterior of particles (Figure 5), consistent with previous observations.^[^ ^9^ ^]^ In agreement with our XRD data (Figure 2, Table 1), EELS data also showed smaller particles (<20 nm) were mostly comprised of Cu(I), indicating they were more oxidized than the larger particles (> 20 nm; mostly Cu(0); Figure 5; Figure S6, Supporting Information). The Cu(0)/Cu_2_O core–shell structure observed has been targeted previously in abiotic NP synthesis experiments due to their desirable properties including that Cu(0)/Cu_2_O core–shell NPs of approximately ≤25 nm (observed here) are stable in air and offer enhanced catalytic properties, e.g., in click chemistry and selective hydroamination.^[^ ^54^, ^55^, ^56^, ^57^, ^58^, ^59^ ^]^ Overall, the data from XRD, TEM‐SAED and TEM‐EELS correspond well and confirm that wild type *S. oneidensis* bioreduced Cu(II), using H_2_ as electron donor, and produced CuNPs predominately in the periplasm of the cell (HyaB cellular location).

### 2.6 Biotechnological Application; Bio‐CuNP Catalysts

**Scheme 1:**
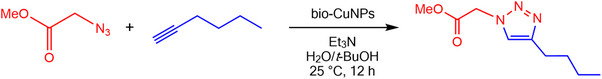
The model click reaction used to confirm catalytic activity of CuNPs biosynthesized. The rection was performed in H_2_O/t‐butanol (15.0 mL, 8:2), at 25 °C with bio‐CuNP catalyst loadings of 0.5, 3, or 6%.

Catalytic activity of CuNPs biosynthesized using H_2_ as an electron donor were tested in a model click reaction, for the cycloaddition of methyl‐azido acetate and 1‐hexyne (**Scheme** **1**). Aliquots of cell suspension containing CuNPs from wild type samples were added to the reaction solution as a catalyst at 3 mol% and 6 mol% Cu. After stirring at 20 °C overnight reactions had full conversion to the triazole product that was confirmed by ^1^H‐NMR (Figure S9, Supporting Information). These findings align with previous work showing *S. oneidensis*‐biosynthesized CuNPs can catalyze the synthesis of a range of triazole derivatives at loadings as low as 1.1%, comparable to chemical Cu catalyst loadings used for triazole production.^[^ ^9^, ^60^, ^61^ ^]^ This further emphasizes the biotechnological potential of harnessing the metabolism of *S. oneidensis*’ to bio‐transform Cu/metal‐laden aqueous wastes into a valuable product, in line with the principals of a circular economy.

## 3 Conclusion

This work shows, for the first time, that [NiFe] hydrogenases can facilitate Cu(II) bioreduction. In wild type (WT) *S. oneidensis MR‐1*, H_2_ oxidation by HyaB was coupled to complete reduction of Cu(II) to Cu(0), leading to formation of CuNPs in the periplasm of cells. Through monitoring hydrogen metabolism, HyaB was shown to be active after Cu(II) exposure, demonstrating resistance to Cu(II) inhibition that could be particularly important in the context of metal biorecovery from Cu‐containing industrial effluents. Additionally, this work also confirmed that OMCs do not play a major role in CuNP synthesis as Cu‐OMC interactions appear to disrupt extracellular electron transfer. CuNPs biosynthesized with H_2_ as electron donor comprised Cu(0) and Cu(I) in a core–shell configuration, with crystal defects such as stacking faults and nanotwinning, resulting in their strong catalytic performance for click chemistry reactions. Overall, this work has advanced understanding of Cu(II)‐bioreduction mechanisms in *S. oneidensis*. This provides a basis for further optimization toward industrial scale‐up and deployment of *S. oneidensis MR‐1* for simultaneous Cu biorecovery and nanocatalyst biosynthesis. More broadly, this work also contributes toward improved understanding of potential mechanisms of Cu biogeochemical cycling in the environment.

### 3.1 Outlook

It is becoming increasingly clear that [NiFe] hydrogenases are important metal reductases in metal‐reducing bacteria, like *Shewanella oneidensis*, catalysing bioreduction of environmentally and technologically important metals including Pd(II), Au(III), Tc(VII), Fe(III), and, now, Cu(II).^[^ ^4^, ^5^, ^29^, ^34^, ^38^ ^]^ To fully harness the potential of these enzymes in whole cell systems at scale, understanding the impact of physicochemical conditions on hydrogenase function is paramount to ensure optimal biorecovery of Cu and/or other metals as nanoparticles. Indeed, industrial feedstocks will undoubtably have both variable/challenging compositions including mixed‐metals and the presence of various counter ions and ligands. It is known that activity of some [NiFe] hydrogenases is tightly controlled by the physicochemical conditions,^[^ ^62^, ^63^, ^64^, ^65^, ^66^ ^]^ but the impact of these parameters specifically on HyaB activity in *S. oneidensis* remains largely unexplored. A good understanding of limitations on HyaB activity can inform bioprocess design and bioengineering approaches to manipulate the physiology of *S. oneidensis*, ultimately to improve metal biorecovery under industrial effluent conditions. Besides this, bioengineering‐based‐enhancements can also be utilized to develop bio‐NP catalysts further, for example, to express enzymic biocatalysts that can be utilized alongside metal bio‐NP catalysts in one‐pot tandem/cascade reactions, e.g.,^[^ ^67^ ^]^ Additionally, we report that Cu(II) can negatively impact on the electron transfer properties of *c‐*type cytochromes like MtrC. These are key metal‐reducing enzymes and understanding impacts of Cu and other metals is vital as it may challenge biorecovery from complex mixed‐metal feedstocks. Deeper exploration of these fundamental metal–cell interactions will also serve to improve understanding of microbial metal‐reducing processes and proteins in environmental settings relevant for contaminated land and water (bio)remediation, and biogeochemical cycling.

## 4 Experimental Section

### Electron Donor and Hydrogenase Deletion Mutant Bioreduction Experiments

*S. oneidensis* bioreduction experiments using different electron donors (lactate, hydrogen) and different hydrogenase deletion mutants alongside wild type *Shewanella oneidensis* MR‐1 (referred to throughout as “WT”), were set up. The following hydrogenase deletion strains were used: JG1454, lacks the periplasmic [Fe, Fe] hydrogenase *hydA*, referred to throughout as *ΔhydA*; JG1455: lacks the periplasmic [Ni, Fe] hydrogenase *hyaB*, referred to throughout as *ΔhyaB*; JG2642: lacks both *hydA* and *hyaB* hydrogenases, referred to throughout as *ΔhydA/ΔhyaB*.^[^ ^68^ ^]^

### Culturing – All Strains

Anaerobic cultures of *S. oneidensis* (wild type and deletion mutants) were grown in defined minimal medium.^[^ ^9^ ^]^ The medium was adjusted to pH 7.4 using NaOH, flushed with N_2_ to remove oxygen, transferred into an anaerobic cabinet (atmosphere 95% N_2_, 5% H_2_), decanted into 100 mL serum vials, sealed with butyl stoppers, and autoclaved at 126 °C. After cooling, sterilized anaerobic medium was inoculated with 1% v/v mid‐log phase culture (grown aerobically in tryptic soy broth) and bottles were incubated overnight (30 °C). Anaerobic cells were harvested and washed in 30 mm bicarbonate buffer by centrifugation (5000 g for 20 min at 4 °C) under anaerobic conditions (N_2_ 80%, CO_2_ 20%, referred to as “N_2_:CO_2_” hereafter). After the final wash, cells were resuspended in ≈10 mL of fresh bicarbonate buffer (30 mm, pH 7) and flushed with N_2_:CO_2_ mix for 20 min prior to use in bioreduction experiments.

### Bioreduction Experiments

Cu(II) bioreduction experiments were performed using standard pre‐grown and washed “resting cell” experiments with wild type *S. oneidensis* MR‐1 and each of the deletion mutant strains. The reaction medium contained anaerobic bicarbonate buffer (30 mm, pH 7, N_2_:CO_2_ purged), CuSO_4_ (100 µm), and either H_2_ or sodium lactate (30 mm) as the electron donor. Washed cells were added anaerobically to reaction bottles, to a final optical density (OD_600 nm_) of 0.4, and incubated at 30 °C. No electron donor and no cell controls were set up in parallel. Periodic samples were taken to monitor changes in aqueous chemistry; 1 mL samples were centrifuged at 14 800 g for 10 min to separate solids (cells and insoluble Cu) and supernatant. To monitor Cu_(aq)_, sample supernatants were diluted and acidified in 2% HNO_3_ for measurement using an Agilent 7500cx inductively coupled – plasma mass spectroscopy (ICP‐MS) instrument. Samples from experiments supplemented with lactate were analyzed using capillary anion exchange chromatography (IC) using a Dionex ICS5000 instrument to quantify the lactate and other organic acid breakdown products. Bathocuproine (BSC) reagent^[^ ^69^, ^70^ ^]^ (6 mm), was used to identify Cu(I) in samples where a small amount of Cu_(aq)_ was removed but CuNPs did not form (i.e., in lactate and no donor controls) and speciation could not be determined by XRD or TEM‐SAED/EELS.

### Hydrogen Generation/Consumption Experiments

To monitor hydrogen metabolism, including H_2_ generation/consumption during Cu(II) bioreduction, washed resting WT cells were added to 20 mL headspace autosampler vials containing 30 mm sodium bicarbonate buffer supplemented with 10 mm sodium pyruvate final OD_600 nm_ = 0.4. The vials were loaded into an Agilent 7697A headspace sampler that sacrificially sampled 1.5 mL of the headspace from each vial, every 15 min for 2 h. Headspace samples were measured using an Agilent 7890A Gas Chromatogram. Sacrificial reaction vials to measure H_2_ consumption (coupled to Cu bioreduction) were also prepared at the same time as the vials used to measure H_2_ generation but were incubated without sampling for 2 h (30 °C) to allow H_2_ to build up in the headspace from pyruvate fermentation via HyaB.^[^ ^35^ ^]^ After the 2 h incubation period, 100 µm CuSO_4_ was added to each vial before loading into the autosampler and repeating the sacrificial sampling regime (every 15 min for 2 h, plus a 20 h sample measured the next day). These sacrificial sampling regimes for H_2_ generation/consumption were repeated three times, each time with freshly prepared reaction vials, to obtain triplicate measurements. A control using the *ΔhyaB* strain was run in parallel.

### Cu(II) Induced Re‐oxidation of MtrC

Cu(II) induced reoxidation of pre‐reduced MtrC was used to explore potential for outer membrane cytochrome mediated Cu(II)‐reduction. Soluble Strep‐tagged MtrC, incorporated in a pBAD202/D‐TOPO vector into *S. oneidensis*, was purified from spent medium following arabinose induction as described previously.^[^ ^71^, ^72^ ^]^ MtrC purity was assessed by denaturing gel electrophoresis, electronic absorbance spectroscopy and LC‐MS. Concentrations of MtrC protein were determined by electronic absorbance of the air‐equilibrated (oxidized) forms using ε_410 nm_ = 1326 mM^−1^ cm^−1^. Purified MtrC was stored in 100 mm TRIS HCl buffer, pH 8 with 150 mm NaCl and frozen as aliquots at −80 °C. The CuSO_4_ induced re‐oxidation of MtrC was performed in anaerobic buffer (100 mm TRIS‐HCl, 150 mm NaCl, pH 8) with 0.9 µm MtrC. Aliquots of sodium dithionite (0.5 mg mL^−1^) were freshly prepared in anaerobic buffer under N_2_ atmosphere. 10 electron equivalents (≈5–10 µL sodium dithionite stock^[^ ^73^ ^]^) were added in 1 µL aliquots as determined by the spectral features of fully reduced MtrC at 421 nm and 552 nm. Re‐oxidation was triggered by the addition of CuSO_4_ stock (100 µm). Spectra were recorded in quartz cuvettes in a Biochrom WPA Biowave II Diode‐array UV/Vis spectrophotometer under an N_2_ atmosphere. Spectra were recorded every 5 s for 3.5 min for full re‐oxidation of MtrC. The percentage of hemes reduced was quantified using the baseline‐corrected absorbance of the reduced heme Soret at 420 nm *minus* oxidised heme Soret at 410 nm. The absorbance at time 0 was taken to be the fully reduced state (100%).

### Pd(II) Bioreduction with Cu(II) Pretreated, Lactate‐Supplemented Cells

Aliquots from lactate‐supplemented Cu(II)‐bioreduction experiment, after 24 h incubation, were spiked with 180 µm Pd (known to be readily reduced by OMCs with lactate as electron donor)^[^ ^17^ ^]^ and monitored for signs of Pd bioreduction (colour change yielding black Pd‐NP formation confirmed by XRD).

### Solids Characterization

All preparation for solids characterization was performed under a N_2_‐atmosphere, using N_2_‐purged reagents.

X‐ray diffraction (XRD) was performed on a Bruker D2 Phaser diffractometer with a Cu Kα x‐ray source (wavelength 1.54 Å). Scans were taken between 2*θ* values 30° and 70°. Solid samples were washed in deionized water, concentrated by centrifugation, and dried directly onto a zero‐background silicon sample holder. XRD analysis used the identified {200} and {220} peaks of Cu_2_O and {111} peaks of Cu(0) to identify the mean crystallite size using the Scherrer equation, FWHM = Kλ / Dcosθ, where FWHM is the full width at half‐maximum of the diffraction peak; k – shape constant; D – crystallite size; θ – Bragg angle. All peaks were consistent with The International Centre for Diffraction Data (ICDD) values for Cu(0) or Cu_2_O.

Whole cell samples for imaging using transmission electron microscopy (TEM) were prepared by drop casting 2 µL of cell/nanoparticle suspensions onto gold grids, with a holey carbon film (200 mesh). For ultrathin section analysis, cell/nanoparticle suspensions were fixed and resin embedded. Fixative solution (2.5% electron microscopy grade glutaraldehyde in 0.1 m HEPES, pH 7.2) was applied to pelleted solids for 2 h at room temperature. The fixative was removed, solids were pelleted (centrifugation) and resuspended in 1.5% low‐gelling temperature agarose before quickly re‐pelleting and setting on ice. Agarose embedded pellets were trimmed, and fresh fixative applied for 1 h. Samples were washed three times in deionized water for 5 min on a rotary shaker. No stain or further fixative was used as reduced osmium/potassium ferrocyanide solution reacted with the Cu to produce imaging artefacts (Section S1, Figure S10, Supporting Information). Samples were dehydrated for 15 min in increasing ethanol solutions (30%, 50%, 70%, 90%, 100%) before washing twice in acetone for 30 min. Samples were infiltrated with resin using increasing graded concentrations mixed with acetone (25%, 50%, 75%, 100%) for ≈12 h per infiltration step. Pellets were transferred to molds with fresh 100% resin and polymerized at 60 °C for 3 days. Ultrathin sections (≈50 nm) were cut with a Diatome diamond knife on a Leica ultramicrotome and were collected on gold TEM grids with a holey‐carbon film (200 mesh). In samples where no CuNPs had formed (*ΔhyaB*), imaging contrast was poor and Pb‐citrate stain was applied directly to thin‐section samples on TEM grids for 10 min in a CO_2_ scrubbed atmosphere.

TEM and high angle annular dark field (HAADF) scanning transmission electron microscope (STEM) imaging was performed on an FEI Talos F200A TEM with an X‐FEG electron source operated at an accelerating voltage of 200 kV. The STEM convergence angle was 10.5 mrad and the HAADF detector angle was 40–200 mrad. Energy dispersive x‐ray spectroscopy (EDX) was recorded in the TEM using an FEI Super‐X detector. Electron Energy Loss Spectroscopy (EELS) was performed on an aberration‐corrected Thermo Fisher Titan G2 STEM operated at 200 kV. HAADF STEM imaging was conducted with a probe convergence angle of 21 mrad, a HAADF inner angle of 55 mrad, and a probe current of ≈70 pA. EELS spectra were collected using a Gatan Imaging Filter Quantum ER system with a 5 × 10^−3^m entrance aperture and an energy resolution of ≈1.8 eV. EDX analysis was performed using the Titan's Super‐X four silicon drift EDX detector system with a total collection solid angle of ≈0.7 srad. Raw EELS data were processed using the HyperSpy package (version 1.7.5); sample spectra were compared to reference spectra and EELS maps for Cu species distributions were calculated by multiple linear least squares (MLLS) fitting.

### Catalytic Activity Testing

A model click reaction was used to confirm catalytic activity of CuNPs synthesized. Briefly, methyl azidoacetate (0.75 mmol), hexyne (0.75 mmol) and triethylamine (0.75 mmol) were dissolved in H_2_O/t‐butanol (15.0 mL, 8:2). An aliquot of washed cell suspension (stored under N_2_ in anaerobic deionized water) containing CuNPs produced via the successful bioreduction of 100 µm Cu(II) with H_2_, was added to the reaction solution as a catalyst at two different loadings, 3 mol% Cu and 6 mol% Cu. The reaction was stirred at 20 °C overnight and ethyl acetate (3×10 mL) was used to extract the organic phase, which was then washed three times with brine (3×20 mL) and dried over anhydrous MgSO_4_. The organic phase was evaporated at room temperature overnight and crude solids were resuspended in d‐chloroform for characterization by ^1^H‐NMR.

## Conflict of Interest

The authors declare no conflict of interest.

## Supporting information



Supporting Information

## Data Availability

The data that support the findings of this study are available from the corresponding author upon reasonable request.
