# Supplementary material for: Hydrogenase Mediated Biosynthesis of Catalytically Active Cu Nanoparticles
Source: Small. 2025 Jul 14;21(35):2500210. doi: 10.1002/smll.202500210 (PMC12410911; doi:10.1002/smll.202500210)
Supplement: Supplementary file 1 — Supporting Information [file SMLL-21-2500210-s001.docx]

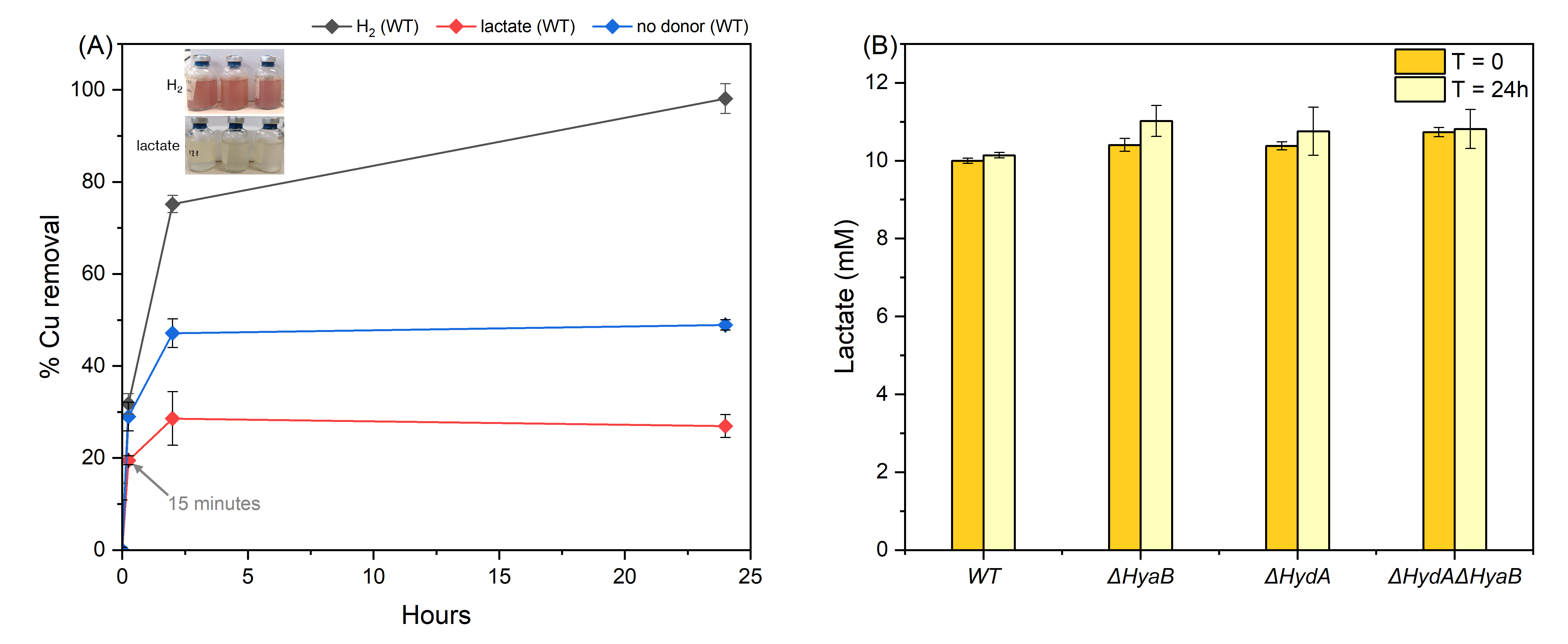


Figure S1. ICP-MS measurement of Cu(II) removal in WT experiments compared to controls (no electron donor, lactate-supplemented). Inset: pictures of H_2_ and lactate supplemented reaction vials after 24 hours. Autoclaved cell controls and no cell controls for both hydrogen and lactate systems were also set up in parallel and no Cu removal was observed in these controls after 24 h. (B) IC measurement of lactate, in lactate supplemented experiments, over 24h for WT and hydrogenase deletion mutant strains.


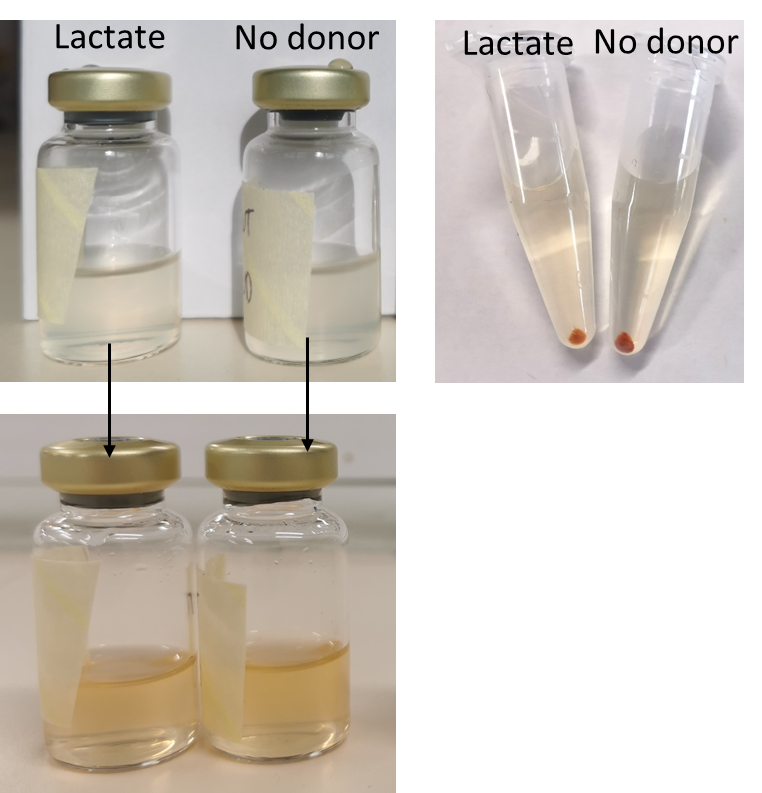


Figure S2. Colour change observed when bathocuproine ligand was added to lactate and no donor controls, after 24 hours incubation with 100 µM Cu(II). Top left: lactate and no donor controls before bathocuproine added, bottom left: the same bottles, after bathocuproine added. Top right: centrifuged aliquots of lactate and no donor controls after bathocuproine addition.


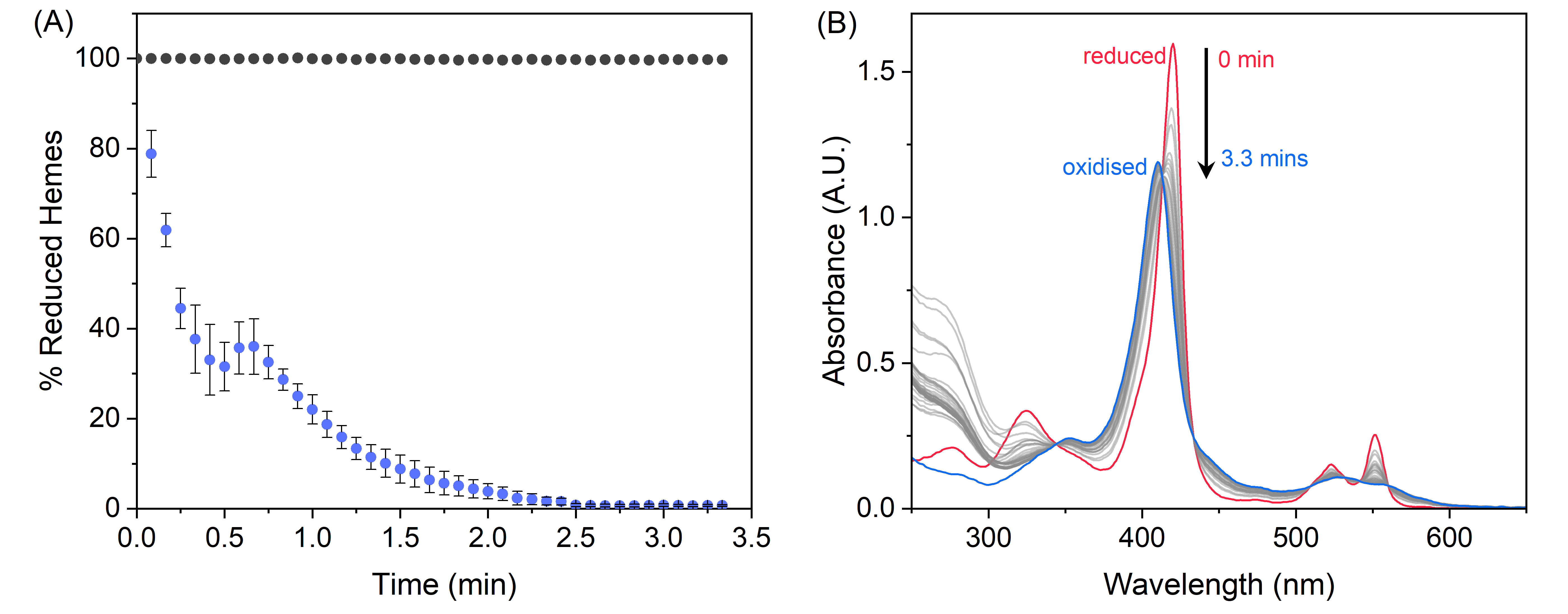


Figure S3. CuSO_4_ (100 µM) induced reoxidation of purified MtrC (0.9 µM) with (A) showing % reduced hemes recorded every 5 seconds for 3.5 minutes with Cu(II) added (blue dots) and no Cu (black dots) and (B) showing the change in absorption spectra, after Cu(II) was added, as the protein is oxidised over the same time period.


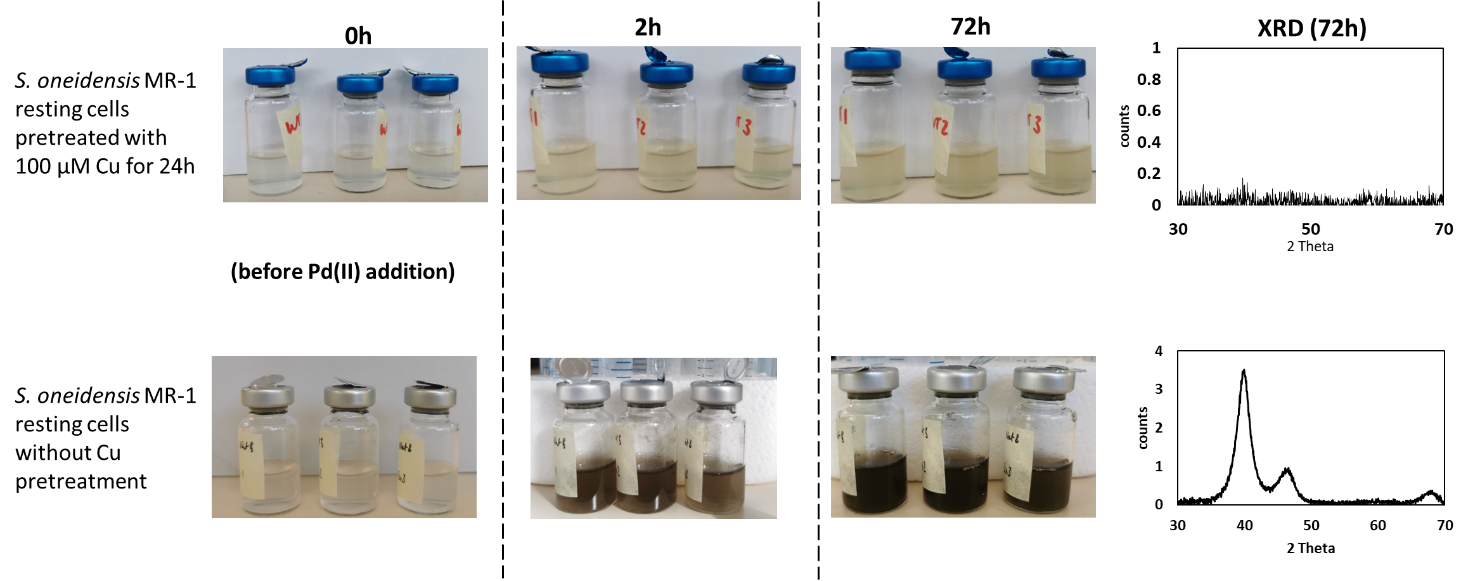


Figure S4. Top row: Cells incubated for 24 hours with 100 µM Cu(II) and 10 mM lactate before being challenged with 180 µM Pd(II)and incubated at 30°C. After Pd(II) addition, samples were checked at 2 hours and again 72 hours incubation. The 72 h samples were washed in deionised water and measured using XRD: no evidence of Pd(II) reduction was observed in Cu treated systems (no colour change to black or Pd-NPs detected by XRD). Bottom row: cells incubated for 24 hours without Cu pretreatment over the same 0, 2 and 72h period. Cells not pretreated with Cu(II) bioreduced Pd(II); colour change to black and characteristic nanocrystalline cubic Pd(0) peaks at 2 theta values of 40, 46, and 68 degrees, corresponding to the {111}, {200}, and {220} planes of Pd(0), respectively


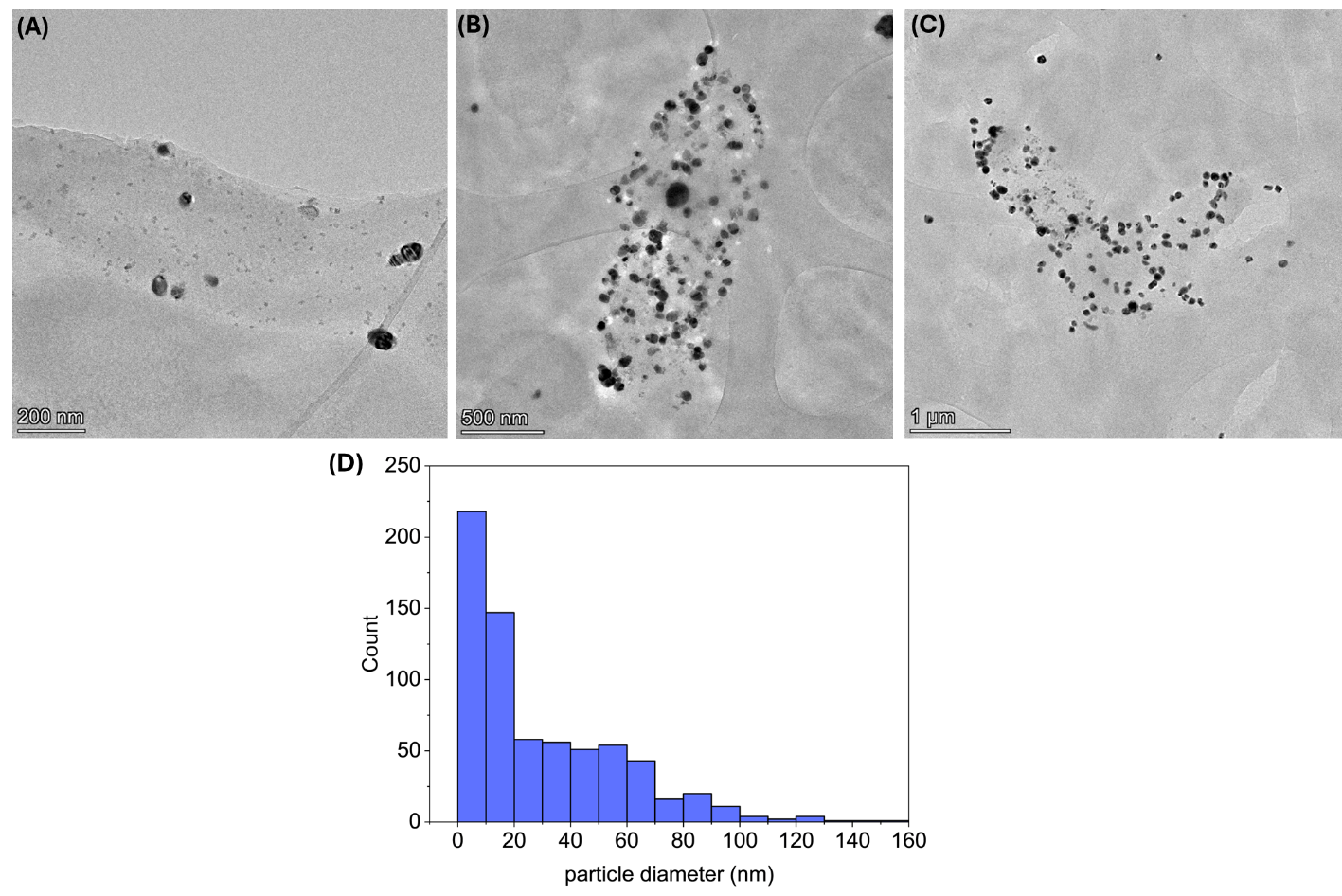


Figure S5. TEM images showing Cu nanoparticles on *S. oneidensis* cells (A-C) and corresponding particle size distribution (D) based on measurement of 680 random Cu nanoparticles.


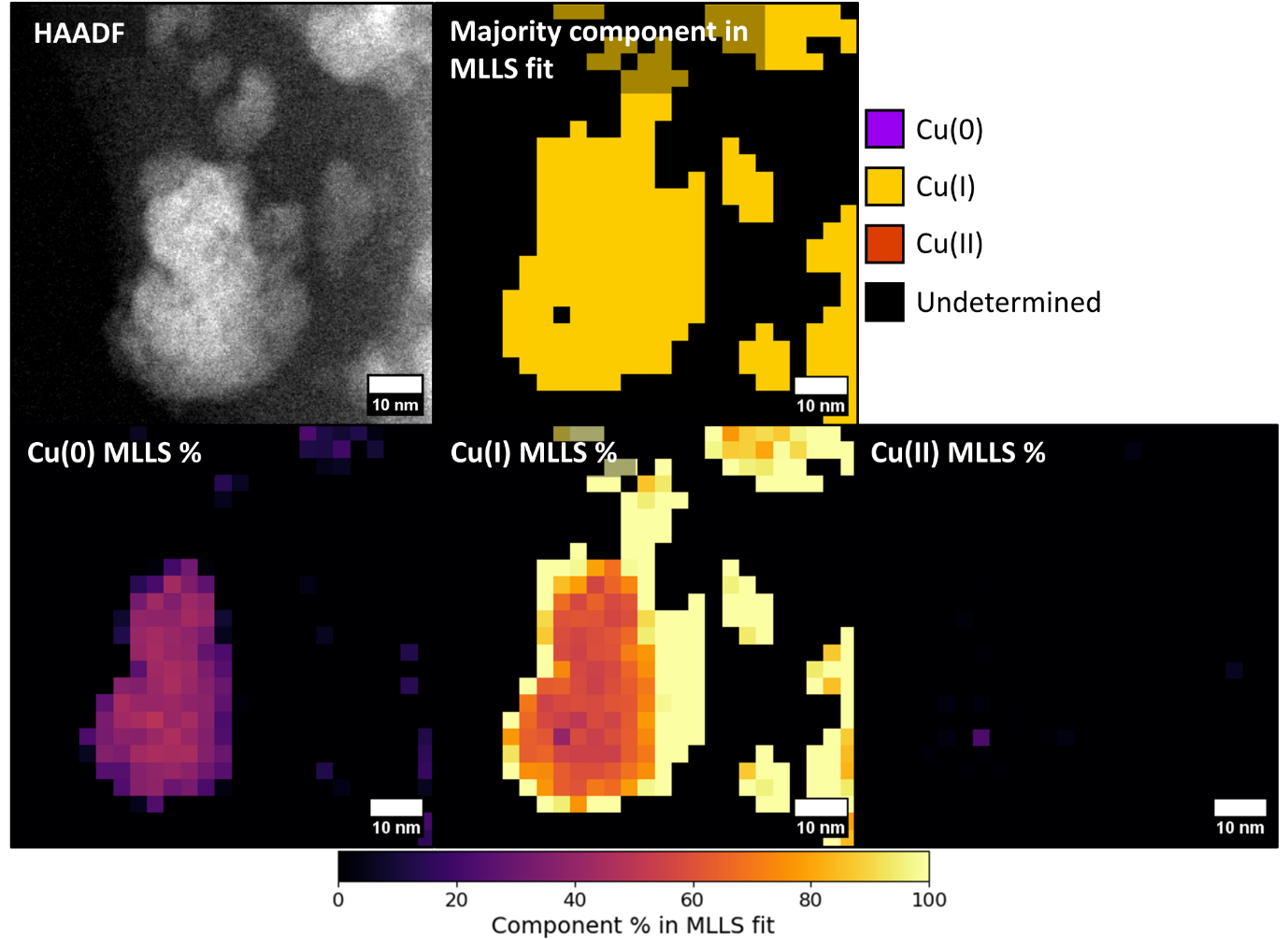


Figure S6. HAADF image CuNPs and corresponding EELS maps showing species distributions calculated by MLLS fitting against reference spectra (shown in Figure S8). Here, it can be seen smaller CuNPs are more oxidised compared to larger CuNPs (Figure 5).


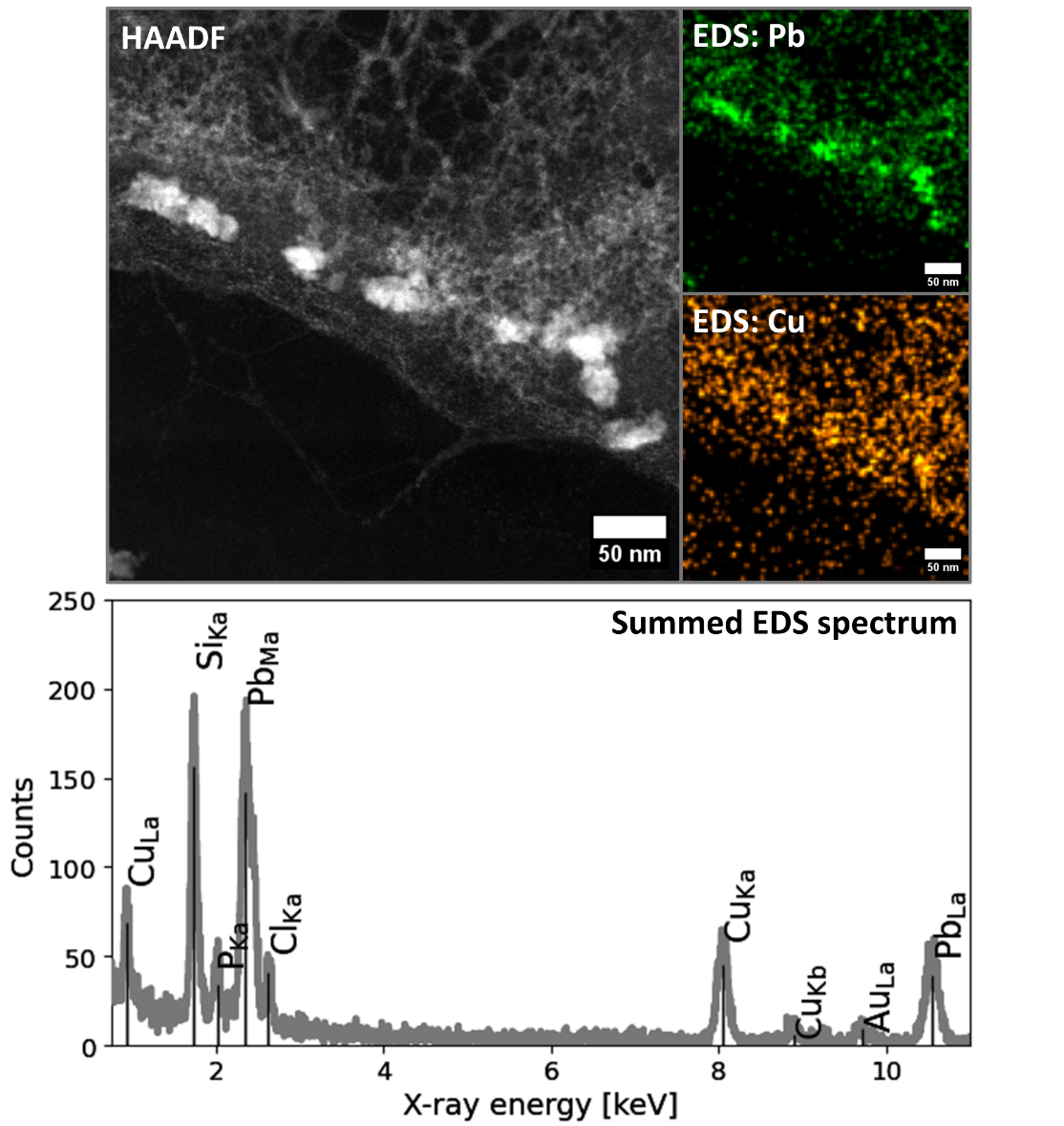


Figure S7. EDS sum spectra, HAADF STEM image and STEM EDS elemental maps from thin-sectioned *ΔhyaB* samples demonstrating that high intensity precipitates are Pb artifacts from Pb citrate negative staining. A definite Cu signal is present from amorphous biosorbed Cu(II) or Cu(I). The weak Au signal in the sum spectra is an artifact from the Au TEM support grid.


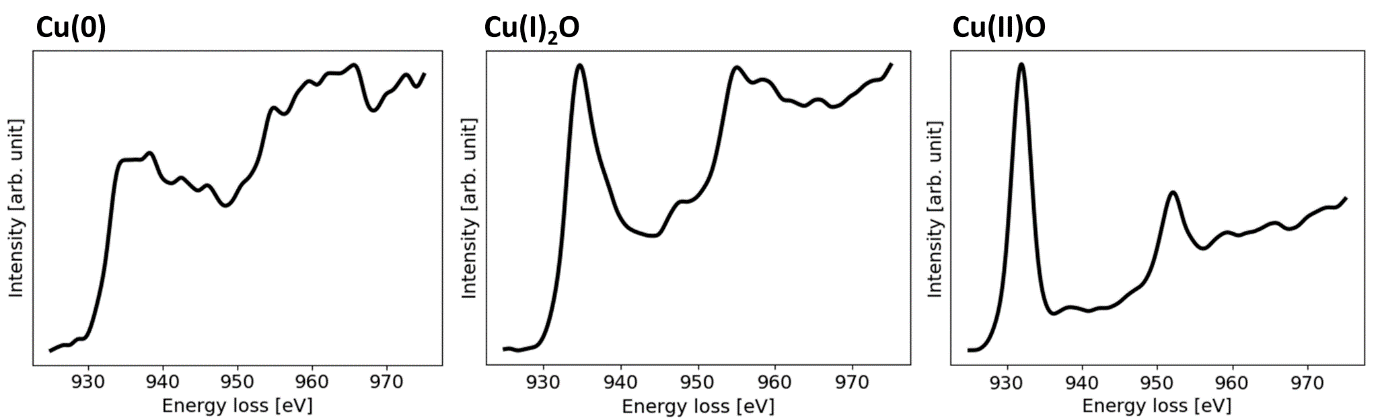


Figure S8. Cu reference electron energy loss spectra used to fit CuNP spectra in Figure 5 References from [1].


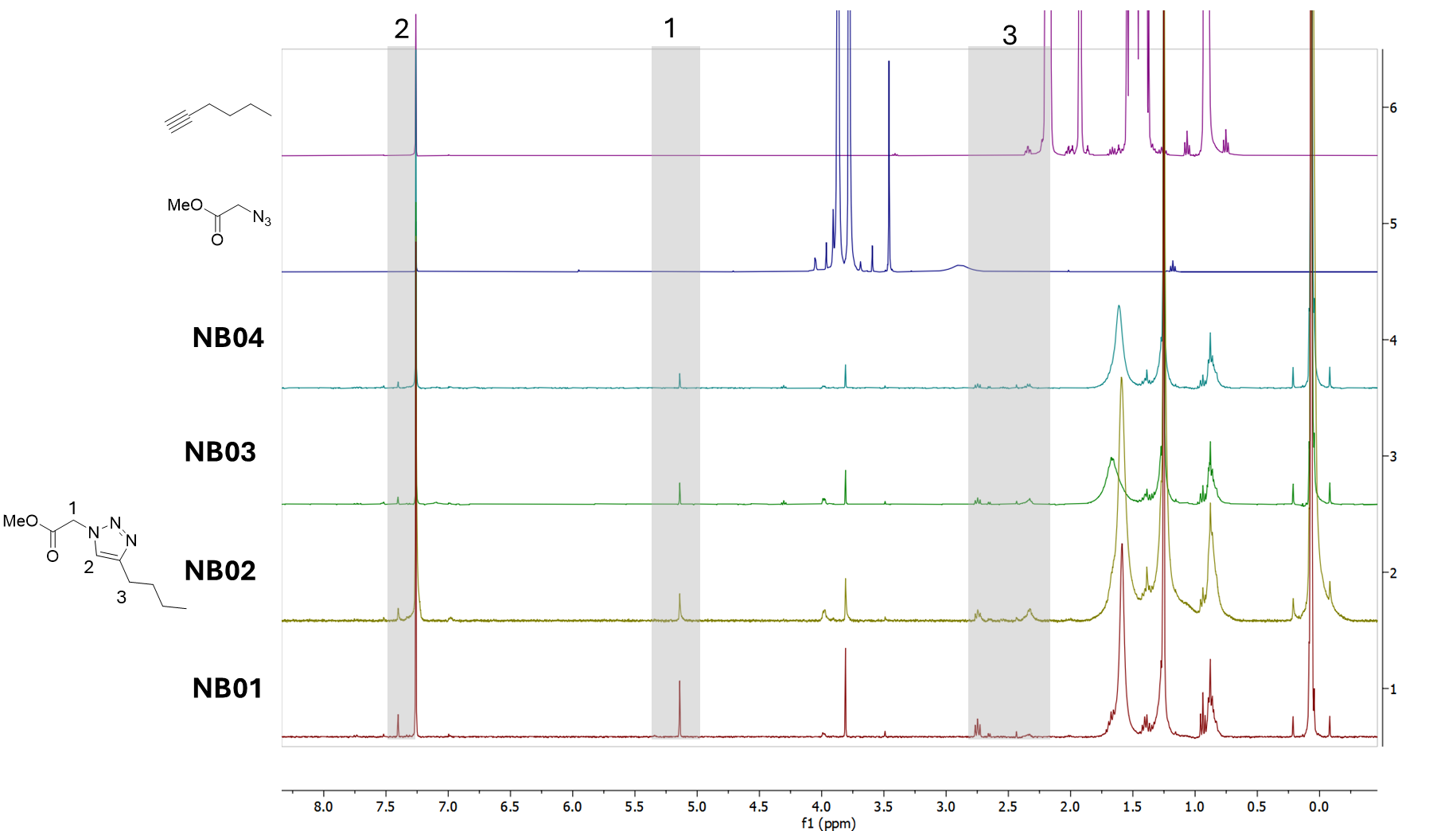


Figure S9. Crude ^1^HNMR spectra from the model Cu-catalysed click reaction (Scheme 1, main text). Loadings: 3 mol % Cu (NB01 and NB03) and 6 mol % Cu (NB02 and NB04. All CuNP catalysts were produced using wild type *S. oneidensis* MR1 with H_2_ as the electron donor. Conversions were calculated using the integrations of the CH_2_ signal of the triazole product and the CH_2_ signal of the azide starting material.

**Section S1. Reduced Osmium Stain interaction with sorbed Cu**

For thin section samples, initial sample preparation comprised a resin embedding protocol that included a reduced osmium staining step (based on Kimber et al., 2018). The reduced osmium stain (1% osmium with 0.75% potassium ferrocyanide in 0.1 M HEPES buffer, pH 7.2), is routinely added to biological samples to improve contrast during TEM imaging. However, when preparing our samples using this stain, they turned blue after staining (Figure S11 and visible artefacts in TEM images of both the WT and *ΔHyaB* samples were noted (Figure S11 Cu-, Fe- and N-rich nanocubes). It seems likely that the potassium ferrocyanide in the stain was reacting with Cu present, as either CuNPs or as sorbed Cu, to produce a copper(II)-cyanoferrate species e.g. copper(II) hexacyanoferrate. Copper(II) hexacyanoferrate, also known as Cu-substituted Prussian blue, has a striking blue colour and is characteristically reported to form nanocubes, like those observed in both samples [2-6].


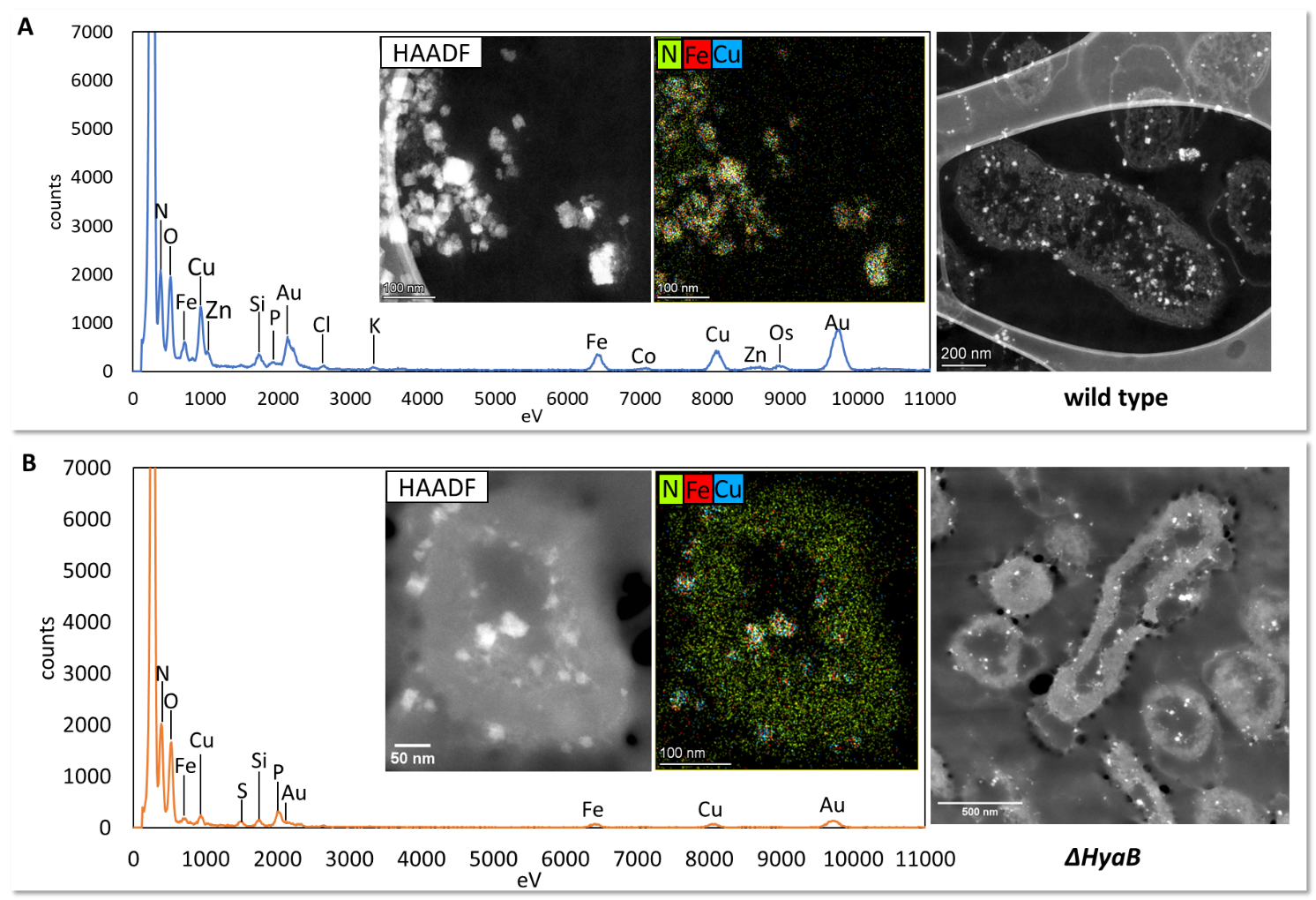


Figure S10. Thin section images and EDS spectra of WT (A) and *ΔhyaB* (B) samples after reduced osmium stain was applied during the resin embedding process. Inset, top panel: Eppendorf tube containing agarose embedded cell pellet from wild type samples, after reduced osmium stain was applied.

References

1. Kimber, R.L., et al., *Copper bioreduction and nanoparticle synthesis by an enrichment culture from a former copper mine.* Environmental Microbiology, 2023. **25**(12): p. 3139-3150.

2. Wessells, C.D., R.A. Huggins, and Y. Cui, *Copper hexacyanoferrate battery electrodes with long cycle life and high power.* Nature Communications, 2011. **2**(1): p. 550.

3. Liu, S., et al., *Copper hexacyanoferrate nanoparticles as cathode material for aqueous Al-ion batteries.* Journal of Materials Chemistry A, 2015. **3**(3): p. 959-962.

4. Baioni, A.P., et al., *Copper hexacyanoferrate nanoparticles modified electrodes: A versatile tool for biosensors.* Journal of Electroanalytical Chemistry, 2008. **622**(2): p. 219-224.

5. Jia, Z., J. Wang, and Y. Wang, *Electrochemical sodium storage of copper hexacyanoferrate with a well-defined open framework for sodium ion batteries.* RSC Advances, 2014. **4**(43): p. 22768-22774.

6. Jain, P., S. Jha, and P.P. Ingole, *Strong metal–support interaction in copper hexacyanoferrate nanocube decorated functionalized multiwall carbon nanotubes for enhanced bi-functional oxygen electrocatalytic activity and stability.* Sustainable Energy & Fuels, 2022. **6**(4): p. 1094-1107.
